# Bone and Joint Tuberculosis: The Experience From a Tuberculosis Department in Northern Greece

**DOI:** 10.1155/crdi/1632733

**Published:** 2025-05-22

**Authors:** Anastasios Vogiatzoglou, Maria Hadji Mitrova, Eleni Papadaki, Maria Sionidou, Katerina Manika

**Affiliations:** Pulmonary Department of Aristotle University of Thessaloniki, General Hospital of Thessaloniki “Georgios Papanikolaou”, Thessaloniki, Greece

**Keywords:** bone tuberculosis, joint tuberculosis, osteoarticular tuberculosis, tuberculous spondylitis

## Abstract

**Introduction:** Tuberculosis (TB) of bones and joints is a relatively rare manifestation of the disease. Biopsy is the key to diagnosing it, while chemotherapy is the cornerstone of treatment. Some patients need surgery in addition to anti-TB drugs. We present a series of eight cases of bone and joint TB.

**Method:** The files of the patients with TB diagnosed and treated at the Pulmonary Department of Aristotle University of Thessaloniki (A.U.Th.) between 2013 and 2022 were reviewed. Patients with a bone or joint infection due to *M. tuberculosis* were selected.

**Cases Presentation:** During these ten years, 307 cases of TB were found. Eight of them were TB of bones and joints (2.6%). Six patients were men and two women, with a mean age of 53.5 years and a standard deviation of 18.2 years. Half of them were native Greeks. The spine was involved in 4 cases, while two of the patients also had pulmonary TB. In seven cases, *M. tuberculosis* DNA was detected by PCR. Chemotherapy with anti-TB drugs was administered to all eight patients, with three of them undergoing surgery in addition to anti-TB medication. The minimum treatment duration was twelve months. Six out of eight cases had a good outcome.

**Conclusions:** TB is a rare cause of infection of bones and joints; however, it should be included in the differential diagnosis of bone lesions. PCR for *M. tuberculosis* seems to have significantly good results in microbiological confirmation of osteoarticular TB.

## 1. Introduction

According to the World Health Organization (WHO), tuberculosis (TB) remains a global threat, the course of which has been significantly affected by the COVID-19 pandemic. It is estimated that 10.6 million people fell ill with TB in 2021, an increase of 4.5% from 10.1 million in 2020. Most of the 6.4 million cases reported in 2021 were pulmonary TB, while 1.1 million cases of extrapulmonary disease were reported [1].

In 2023, Greece reported a TB incidence rate of 4.71 cases per 100,000 population, marking a 54% increase compared to 2022. Greece is classified as a low-incidence country by the WHO, but underreporting may affect the estimated burden of the disease. The upward trend in TB cases has been observed in both Greek-born and foreign-born populations, with a notable increase among individuals from countries with high TB incidence [2].

TB of the bones and joints (osteoarticular) accounts for 1 to 2% of TB cases [3]. Some common localizations of the disease are the spine, hip, knee, limb, elbow, hand, and shoulder. Patients are generally young men, immigrants from countries where the disease is endemic. The symptoms are nonspecific, mainly pain and swelling locally, whereas the typical symptoms of TB are rare. Diagnosis is usually delayed due to the slow-progressing nature of the disease and low clinical suspicion in areas with a low incidence of TB. Osteoarticular TB should be suspected in persistent back pain in patients at high risk for TB, such as migrants and immunosuppressed individuals [4–9].

Spinal TB, also known as Pott's disease, is the most common form of osteoarticular TB, representing almost half of the cases. The thoracic spine is mostly affected. Imaging can be suggestive, but there are no pathognomonic findings. Computed tomography (CT)–guided biopsy is commonly used to obtain samples [4–9].

Biopsy plays a key role in diagnosis. A sample should be obtained for histopathological and microbiological examination. Culture is the Golden Rule for diagnosing TB and is considered necessary to determine the sensitivity to anti-TB medication. Its disadvantage is the time required for the development of *M. tuberculosis*. However, molecular methods for detecting it are now available. GeneXpert MTB/RIF is based on polymerase chain reaction (PCR). It allows rapid detection of *Mycobacterium* DNA in a few hours and has a sensitivity of 77%–90% in tissue samples. It is recommended by the WHO for diagnosis from nonrespiratory specimens in patients with suspected extrapulmonary TB [6, 10–13].

Chemotherapy with anti-TB drugs is the cornerstone in the treatment of bone and joint TB. It is administered regardless of whether there is a need for surgery. Anti-TB drugs show excellent penetration into the osteoarticular localization of TB. According to American Thoracic Society (ATS) Guidelines, six- to nine-month regimens containing rifampicin are recommended. However, due to difficulties in assessing the response to treatment, most specialists worldwide tend to extend the regimens to twelve months [4, 14, 15].

Regarding surgical treatment, there are absolute and relevant indications. The absolute ones include neurological deficits, large abscesses with symptoms, kyphosis, and instability of joints and spine. The relevant ones include the inability to diagnose with other techniques, persistent pain, spasticity, and deformity [4–8].

## 2. Methods

The case files of the TB patients diagnosed and treated during the period between 2013 and 2022 at the Pulmonary Department of Aristotle University of Thessaloniki (A.U.Th.), General Hospital of Thessaloniki “Georgios Papanikolaou,” were reviewed. Patients with the diagnosis of osteoarticular TB were selected, regardless of the diagnosis method. Characteristics such as sex, age, origin, medical history, site of bone TB, symptoms, lung involvement, microbiological confirmation, duration of treatment, and surgery were recorded. Outcomes were defined according to WHO guidelines [16].

## 3. Case Presentation

Of the 307 TB cases diagnosed between 2013 and 2022, eight patients with bone and joint TB were identified, i.e., 2.6%. Six of them were men and two were women, with a mean age of 53.5 ± 18.2 years. Four of them were native Greeks, two came from Somalia, one from Ukraine, and one from Georgia. Three of the patients had no other comorbidities, while five of them had at least one factor in their medical history. Patients' characteristics are presented in Table 1.

**Figure 1:**
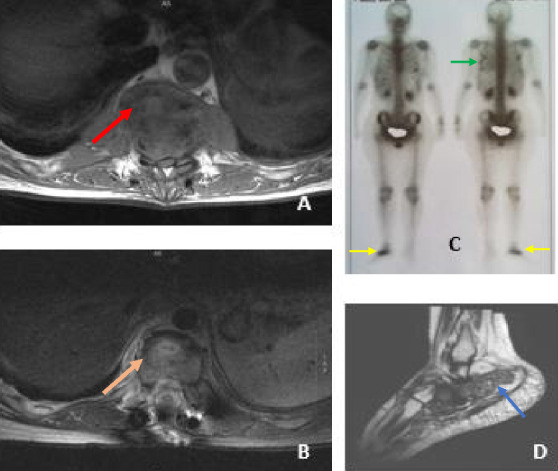
(A, B) MRI of Case 8 showing thoracic tuberculosis before (red arrow) and 12 months after treatment initiation-improvement (orange arrow). (C) Gallium scan of Case 3 showing TB of the ribs (green arrow) and right ankle joint (yellow arrows). (D) MRI of Case 3 showing ankle joint TB involvement (blue arrow)—same case as in (C).

Six out of eight patients had pain as their main symptom, depending on the affected bone or joint. Patient 2 was investigated for fever, Patient 4 had paraplegia in addition to pain, Patient 5 had a swollen hip, and Patient 6 had reduced leg mobility but no paraplegia. Patients 3 and 6 had respiratory symptoms in addition to pain.  Figure 1 shows imaging of TB of the thoracic spine and ankle joint-ribs.

A biopsy was performed in all cases, and a bone or joint sample was sent for microbiological confirmation. Five of the patients were diagnosed with tuberculous spondylitis (Pott's disease). In all but one case, there was microbiological detection of *M. tuberculosis* DNA with a positive PCR method (Xpert Mtb/Rif). In five of these seven cases, *M. tuberculosis* was isolated in culture, while in two it was not. In Patient 7, a histological diagnosis was made. Multidrug-resistant (MDR) *M. tuberculosis* was isolated in one patient. Two patients had pulmonary involvement, which was confirmed by microbiological examination of the sputum.

The type of treatment and outcome are presented in Table 2. Chemotherapy with anti-TB drugs was administered to all patients. Five of them underwent only conservative treatment (62.5% of cases), while the remaining three (37.5% of cases) required surgery in addition to anti-TB medication. Patient 1 had functional problems with the radiocarpal joint, Patient 4 had spinal compression and a neurological deficit, and Patient 5 required drainage of a cold abscess. Patient 1 received 30-month treatment due to multidrug-resistant tuberculosis (MDR-TB), while Patient 3 had resistance to isoniazid and developed several side effects due to anti-TB medication, mainly from the gastrointestinal system, resulting in 20 months of treatment. Patient 5 received a 14-month treatment due to small periarticular effusions. Patient 2 died 1 month after treatment initiation. Patient 6 was lost to follow-up. The remaining 3 patients with TB due to sensitive strains received 12 months of medication.

Most cases (six out of eight) had a good outcome (five completed treatment and one cured). Patient 6 was lost to follow-up, while Patient 2 died due to complications of his comorbidities.

## 4. Discussion

The percentage of osteoarticular TB among all TB patients in the period 2013–2022 in our center was 2.6%, which agrees with the results of a Greek retrospective study from 2011 to 2019 [17]. In contrast to the study of Karabella et al., half of our patients were Greek citizens, while the younger ones were migrants [17]. In the large case series already published in the literature [17–23], a great heterogeneity regarding age was observed, ranging from 31 to 63 years, which is compatible with our results. Similar to our findings, men outnumbered women in several studies [17–19, 21, 23], while this was not the case in some others [20, 22]. As in our study, in the case series of Vielgut et al. [18], most patients were natives, while in the studies of McGuire et al. [23] and Karabella et al. [17], most patients were migrants.

The most affected bone in our case series was the thoracic spine. Part of the literature [18, 21] also found that the spine is the most affected location, while the series of Polley and Dunn [22] found that the thoracic spine is mostly affected. In contrast, Karabella et al. [17] found that the lumbar spine was the most affected site. Two of the patients (25%) had concurrent pulmonary TB. There is great heterogeneity in the studies about this matter. In some of them [23, 24], pulmonary involvement was low (13%–16%), while in others [17, 18, 21], almost half of the cases had concurrent lung TB (42%–47%). The fact that in five of seven cases with positive Xpert, *M. tuberculosis* was developed in culture, while in two it was not, highlights the important role of PCR methods in the diagnosis of osteoarticular TB. High positive rates of PCR were also found in the case series of Vielgut et al. [18] and Dey et al. [24].

In all studies reviewed [17–25], every patient with bone and joint TB received chemotherapy, which was also the case in our patients. In most cases [17–25], patients with bone and joint TB received medication for 9–12 months, which agrees with our findings. In difficult cases, such as those of resistant TB, prolonged treatments, such as those received by Patients 1 and 3, are described. The percentage of surgery treatment in addition to chemotherapy varies in the literature, from 7.5% to 64.7% [17, 18, 21, 23].

Most of our cases (six out of eight) had a good outcome (treatment completed or cured), which agrees with most of the case series in the literature [17–25]. The only death observed in our series was in a patient with sacral TB. The patient had severe underlying conditions, and it was attributed to his comorbidities. Case reports of successful treatment of sacral TB have been reported [26, 27].

## 5. Conclusion

TB is a rare cause of bone and joint infections. Most cases suffered from pain in the affected area and movement disorders. The molecular method of DNA detection of *M. tuberculosis* seemed particularly reliable in this case series, as it gave a positive result even in the two cases where the culture was negative. This highlights the importance of this study in enhancing the use of molecular diagnostic methods in osteoarticular TB. Most patients responded well to anti-TB treatment, whether they needed surgery or not.

## Figures and Tables

**Table 1:** Demographic characteristics, risk factors, detection, pulmonary involvement, biopsy, and microbiological confirmation.

Case	1	2	3	4	5	6	7	8
Sex	Male	Male	Female	Male	Male	Male	Female	Male
Age	78	71	43	47	74	30	58	29
Origin	Greek	Greek	Georgia	Ukraine	Greek	Somalia	Greek	Somalia
Risk factors/medical history	Hypertension, former pulmonary TB	Atrial fibrillation, chronic obstructive pulmonary disease, pulmonary hypertension	None	Hepatitis C, chronic renal failure	Hypertension	None	Diabetes mellitus, breast cancer	None
TB detection	Radiocarpal joint	Sacrum	Ankle joint and ribs	Thoracic spine	Hip abscess	Thoracic spine	Thoracic spine	Thoracic/lumbar spine
Pulmonary involvement^∗^	No	No	Yes	No	No	Yes	No	No
Biopsy^∗∗^	Surgery material	Diagnostic	Diagnostic	Diagnostic	Surgery material	Diagnostic	Diagnostic	Diagnostic
XPERT MTB/RIF	Positive	Positive	Positive	Positive	Positive	Positive	Not done	Positive
Cultivation	Positive	Positive	Positive	Negative	Positive	Negative	Not done	Positive
Mtb sensitivity	MDR	Sensitive	Isoniazid resistance	Sensitive	Sensitive	Sensitive	Sensitive^∗∗∗^	Sensitive
Imaging	Cold radiocarpal abscess on MRI scan	Abnormal uptake of sacrum in PET scan	Abnormal uptake of ankle joint and ribs in PET scan	Lytic foci of thoracic vertebrae on CT scan	Cold hip abscess on MRI scan	Lytic foci of thoracic vertebrae on CT scan	Lytic foci of thoracic vertebrae on CT scan	Lytic foci of thoracic and lumbar vertebrae on CT scan

^∗^Microbiologically confirmed.

^∗∗^Patients 1 and 5 first underwent surgery and then were diagnosed with TB from the tissue that was removed. Patient 4 was first diagnosed with TB through CT-guided biopsy and then underwent surgery.

^∗∗∗^Patient 7 had a histological diagnosis; she received the classic four-drug regimen, with complete remission of symptoms.

**Table 2:** Treatment.

Case	Surgery	Duration of medication (months)	Outcome
1	Radiocarpal joint arthroplasty and fistula removal	30	Treatment completed
2	No	Died 1 month after treatment initiation	Died
3	No	20	Cured^∗∗∗∗^
4	Spinal fusion	12	Treatment completed
5	Abscess drainage	14	Treatment completed
6	No	8	Lost to follow-up
7	No	12	Treatment completed
8	No	12	Treatment completed

^∗∗∗∗^The term cured is used because this patient had also pulmonary TB. She completed treatment as recommended, with evidence of bacteriological response and no evidence of failure.

## Data Availability

Readers can access the data needed to support the conclusions of this article through Tables 1 and 2.
